# The need for a new keyword — “Trial registry-metaresearch” — to track certain uses of clinical trial registry records

**DOI:** 10.1186/s13063-023-07231-1

**Published:** 2023-03-14

**Authors:** Gayatri Saberwal

**Affiliations:** Institute of Bioinformatics and Applied Biotechnology, Biotech Park, Electronics City Phase 1, Bengaluru, 560100 Karnataka India

**Keywords:** Clinical trial registries, Keyword, Systematic review

## Abstract

Public clinical trial registries contain a large amount of information about a large number of trials. Academic researchers have conducted various analyses using such data. However, some of these studies do not concern the medical condition or intervention that is the focus of each trial. We list examples of publications that have performed such analyses. Currently, there is no keyword to track relevant publications. Here, we propose a novel keyword, “Trial registry-metaresearch”, that could be used in such publications. This would be a great help to researchers who wish to more systematically search the literature for such metaresearch.

A public clinical trial registry holds the details of a large number of clinical trials. The World Health Organization recognizes almost 20 of these registries, which are hosted by various countries. Between them, these registries host several hundred thousand records [1], with the largest, ClinicalTrials.gov of the USA, hosting almost 440,000. Although these records have been the subject of many diverse analyses, there is no systematic way to search for publications that capture such metaresearch. Here, I propose a new keyword that would aid researchers to do so.

A government may wish to have quantitative information of the use of the nation’s public clinical trial registry thus far. Other organizations that wish to support a registry may also find this information useful. Such funders may be interested in the number of users who access the registry and the number of pages viewed per day (https://clinicaltrials.gov/ct2/about-site/for-media)[2], or the categories of people who access it (https://clinicaltrials.gov/ct2/about-site/for-media), for instance. Another angle would be to know how scholars have used the database. Elsewhere, I have pointed out that registries have been used for multiple types of analyses, relating to the dissemination of information, tracking scientific discoveries or biomedical applications, and so on [3]. In Table 1, I list one example of each of these various uses of such a registry. Each of these publications reports on metaresearch conducted across trial records in a registry, of a particular piece of information that is not related to a medical condition or intervention, for instance. That is, the analyses are independent of the specific nature of the trial that the record captures.

**Table 1:** Examples of various types of analyses performed with trial registry data, and a sample publication of each. The keywords linked to each publication are also listed

Category of information, and example with reference	Keywords
**1. Reporting quality** A study of almost 800 trials showed that the methods were better reported in trial registry records than in the related publications [4]	Randomized trials; CONSORT statement; Bias; Research design; Prospective trials registration; ICMJE
**2. Research overview** An analysis of almost 400 Expanded Access and Compassionate Use programs characterized the sponsors, enrollment eligibility, fraction of interventions that subsequently received approval by the US Food and Drug Administration approval, etc. [5]	Compassionate use, Expanded access, Experimental drugs, Access to medicines, Ethics, Bioethics, Policy, Right to Try Laws, 21st Century Cures Act, Pharmaceutical industry, Real-world evidence
**3. Research prioritization** An analysis of over 3300 trials conducted in India demonstrated that there were areas in which trials have not been conducted [6]. Such analyses point to research gaps that need to be addressed	Clinical trials, disability‐adjusted life‐years, India
**4. Audit** A study of almost 13,000 trials compared the number and nature of trials that were sponsored by each of the National Institutes of Health. This enabled each institute to assess how much of its own research had reached the stage of clinical trials, compared to that of other institutes. [7]	Clinical trials, ClinicalTrials.gov, National Institutes of Health, clinical trial funding, clinical trial registration, National Institutes of Health Institutes and Centers
**5. Assessment of bias** There has been large-scale non-reporting of the results of trials, and various steps have been taken to rectify this situation, leading to positive change [8]. Not only does such non-reporting constitute research waste, but it could lead to a bias in the literature if positive outcomes are preferentially reported, and could also lead to a breakdown in trust between trial participants and the trial ecosystem, potentially damaging future participation	Clinical trials, Publications, Device approval, United States Food and Drug Administration
**6. Regulatory compliance** One analysis of almost 3700 trials looked into whether trials have broken the law that required them to be registered in a particular registry [9]	Keywords submitted with the manuscript, though not available on the published paper: ClinicalTrials.gov; Clinical Trials Registry—India; hidden duplicates; duplicate registration; registry integrity; secondary ID; Clinical trial registry
**7. Research waste** A study of over 2500 trials showed that over 480 of them were terminated (prematurely) or had enrolled an inadequate number of participants. [10]. There are economic implications if trials are terminated before completion. These costs are both directly related to the trial, and to the opportunity cost of not conducting other trials	Medical ethics, research ethics, clinical trials, trial accrual, recruitment
**8. Global trial landscape** One analysis characterized over 6000 trials that ran in non-high-income countries [11]. The government of a developing nation may wish to know the nature of trials conducted in its territory in comparison to those conducted elsewhere	Clinical trials, Global health, Pharmaceutical companies
**9. Journalology** Authors reporting on their work are often supposed to follow guidelines that the editors of major journals announce, or ratify. An analysis of over 16,600 trials demonstrated that journals may not enforce these guidelines effectively, leading to lower quality publications [12]	CONSORT, Endorsement, Reporting guideline, Completeness of reporting

These categories of analysis - although sometimes with different wording - were outlined in an earlier commentary by the author [3]

However, there is no systematic way to search for publications that do such metaresearch. Even though all the publications listed above are examples of a certain type of analysis, there is no common keyword. In fact, of the 49 keywords, only three occur twice, and one occurs five times.

Therefore, I hereby propose a novel keyword, “Trial registry-metaresearch,” that would apply to studies of this type. Although possible tags include “Trials’ analysis,” “Across trials,” and “Registry analysis,” none of these keywords imply the exclusion of studies that concern a particular condition or intervention.

In a search on PubMed on 4 March 2023, the terms “trial registry-metaresearch” and “trial registry metaresearch” yielded 262,514 and four hits respectively. The first of these numbers is overwhelmingly large and the second is clearly inadequate. Putting the terms in quotation marks for the search did not solve the problem. This reflects the need to formally define the keyword. Just as the keywords in Table 1 are author-provided, I believe that researchers should submit the new keyword with their manuscripts. However, that will not be enough since even if interested colleagues help to spread the word through Twitter and mailing lists for instance, it will take time for the idea of the keyword to spread in the research community.

Although the proposed keyword cannot be included in The Medical Subject Headings (MeSH) thesaurus, which is used to index articles that are listed in PubMed, the staff of the Index Section of the Bibliographic Services Division of the National Library of Medicine (in the USA), work to improve the quality of searches [13]. I intend to be in touch with these staff about the proposed keyword. They would probably have a way to link this keyword to relevant publications even if the keyword has not been submitted by the authors.

In summary, I have proposed that a novel keyword is required to describe metaresearch of clinical trial registry records that do not pertain to a given medical issue or intervention. I believe that the consistent use of such a keyword would benefit those who wish to evaluate the many uses to which registry data has been put. It would also benefit researchers who wish to more systematically search the literature for what metaresearch has, or has not, been carried out.

## Data Availability

Not applicable.
